# Editorial: Ehlers-Danlos syndrome: from bedside to bench

**DOI:** 10.3389/fgene.2024.1399386

**Published:** 2024-04-26

**Authors:** Tomoki Kosho, Shujiro Hayashi, Ken-ichi Matsumoto, Delfien Syx, Anupriya Kaur

**Affiliations:** ^1^ Department of Medical Genetics, Shinshu University School of Medicine, Matsumoto, Japan; ^2^ Department of Dermatology, Dokkyo Medical University, Mibu, Japan; ^3^ Department of Biosignaling and Radioisotope Experiment, Interdiscipnary Center for Science Research, Organization for Research and Academic Information, Shimane University, Matsue, Japan; ^4^ Department of Biomolecular Medicine, Center for Medical Genetics Ghent (CMGG), Ghent University Hospital, Ghent, Belgium; ^5^ Medical Genetics, Advanced Pediatric Center, PGIMER, Chandigarh, India

**Keywords:** Ehlers-Danlos syndrome, heritable connective tissue disorders (HCTD), clinical, pathophysiological, collagen, delineation

The Ehlers-Danlos syndromes (EDS) comprise a genetically heterogeneous group of heritable connective tissue disorders, clinically characterized by skin hyperextensibility, joint hypermobility, and tissue fragility. The currently used nomenclature and classification, published in 2017, recognizes 13 types (Malfait et al., 2017), and several additional types have also been described (Malfait et al., 2020). The pathomechanisms of EDS include abnormalities in the genes for fibrillar collagen types I, III, and V; enzymes modifying or processing these collagens; enzymes involved in the biosynthesis of the glycosaminoglycan chains of proteoglycan; and proteins playing more complex roles in the maintenance of extracellular matrices. Furthermore, clinical overlaps among cases with different types as well as discoveries of EDS-like phenotypes with novel pathomechanisms have been described. The spectrum of EDS is considered to be much wider than expected, and it would help delineate this clinical and pathophysiological spectrum to describe and share clinical and pathophysiological findings of variable and valuable cases.

The scope of this Research Topic (https://www.frontiersin.org/research-topics/31359/ehlers-danlos-syndrome-from-bedside-to-bench/magazine) is to make clinical and pathophysiological delineation of the wide and complex spectrum of EDS. In total, 10 reports are published including four original research articles, five case reports, and one mini review.

Four reports attempted clinical and pathophysiological delineation of vascular EDS (vEDS), the most severe subtype of EDS. Ishikawa et al. described detailed ultrastructural findings as well as clinical features in patients with vascular EDS. Irregularity in the size of collagen fibrils was suggested in 27 patients with vEDS, and the variation tended to be lower in those with less serious vascular complications (Ishikawa et al.). Wei et al. described an association between digestion tract events and non-glycine missense variants from a Chinese family (Wei et al.). Hayashi et al. described a mildly affected Japanese patient found to be caused by a unique in-frame duplication variant in *COL3A1* without alteration in the [Gly-X-Y] triplet repeat sequence of type Ⅲ collagen (Hayashi et al.). Angwin et al. introduced the first experience of pre-implantation genetic diagnosis with surrogacy in vEDS, which could provide a choice for women with vEDS wishing to have unaffected biological children without pregnancy/delivery-related critical events but also provide necessity of careful discussion considering hypothetical and potential risks of all relevant procedures based on ethical, legal, and social backgrounds of each community (Angwin et al.).

Two reports were about classical-like EDS caused by variants in the tenascin-X (TNX) gene (*TNXB*) (clEDS type 1). Yamaguchi et al. established a custom next-generation sequencing-based screening system for this subtype, and provided comprehensive clinical and molecular delineation of this subtype from a cohort comprising nine patients (Yamaguchi et al.). *TNXA*-derived variations were found in >75% and all patients had gastrointestinal complications including perforation, diverticulitis, gastrointestinal bleeding, intestinal obstruction, rectal/anal prolapse, and gallstones (Yamaguchi et al.). Okuda-Ashitaka and Matsumoto made a comprehensive review of experimental studies about TNX using TNX-deficient (*Tnxb* ^*−/−*^) mice; they introduced mechanical allodynia in *Tnxb−/−* mice, inhibited by the anticonvulsant drug gabapentin and a mu-opioid agonist but not by a NSAID indomethacin, and also introduced studies suggesting various roles of TNX (e.g., tumor suppression, epithelial wound healing, liver fibrosis) (Okuda-Ashitaka and Matsumoto).

Two reports are about *AEBP1*-related EDS (classical-like EDS [clEDS] type 2), which represents the 14^th^ subtype following the 13 subtypes according to the 2017 international classification of EDS (Malfait et al., 2017). Angwin et al. (Angwin et al.) and Yamaguchi et al. (Yamaguchi et al.) described additional patients with clEDS type 2; hair loss was recognized as a characteristic feature of this subtype, and importance of a few but critical complications such as vascular or bowel events was also stressed.

The other two reports were about periodontal EDS (pEDS). Angwin et al. described non-oral manifestations in 21 adult patients with periodontal EDS from two United Kingdom cohorts. Easy bruising, pretibial plaques, and brain white matter abnormalities were observed in more than 80% (Angwin et al.). Liao et al. reported the first transcriptomic analysis of patient-derived cells of periodontal EDS. Differential gene expression was found only in monocytes but not in gingival fibroblasts; genes in such biological processes as neutrophil-mediated immunity, response to bacterium, and TNF-α and IL-17 pathway were enriched; and disease ontology enrichment analysis suggested enrichment of genes related to periodontal host defense, inflammatory response, skin disease, and vascular development (Liao et al.).

All these reports are expected to make a substantial contribution in understanding complex clinical and pathophysiological characteristics of various subtypes of EDS. Hopefully, based on these evidences, international collaborative clinical and pathophysiological investigations will be conducted to make a further delineation of each subtype, to provide evidences in establishing better management of patients as well as to develop etiology-based therapies.
